# Multi-modal imaging of tumor cellularity and Tryptophan metabolism in human Gliomas

**DOI:** 10.1186/s40644-015-0045-1

**Published:** 2015-08-06

**Authors:** Jeong-Won Jeong, Csaba Juhász, Sandeep Mittal, Edit Bosnyák, David O. Kamson, Geoffrey R. Barger, Natasha L. Robinette, William J. Kupsky, Diane C. Chugani

**Affiliations:** Departments of Pediatrics and Neurology, Wayne State University School of Medicine, 3901 Beaubien St., Detroit, MI 48201 USA; PET Center and Translational Imaging Laboratory, Children’s Hospital of Michigan, Detroit, MI USA; Karmanos Cancer Institute, Detroit, MI USA; Departments of Neurosurgery and Oncology, Wayne State University School of Medicine, Detroit, MI USA; Department of Neurology, Wayne State University School of Medicine, Detroit, MI USA; Department of Radiology, Wayne State University School of Medicine, Detroit, MI USA; Department of Pathology, Wayne State University School of Medicine, Detroit, MI USA

**Keywords:** Glioma, Diffusion weighted imaging, Positron emission tomography, Amino acid, Cellularity

## Abstract

**Background:**

To assess gliomas using image-based estimation of cellularity, we utilized isotropic diffusion spectrum imaging (IDSI) on clinically feasible diffusion tensor imaging (DTI) and compared it with amino acid uptake measured by α[^11^C]methyl-L-tryptophan positron emission tomography (AMT-PET).

**Methods:**

In 10 patients with a newly-diagnosed glioma, metabolically active tumor regions were defined in both FLAIR hyperintense areas and based on increased uptake on AMT-PET. A recently developed independent component analysis with a ball and stick model was extended to perform IDSI in clinical DTI data. In tumor regions, IDSI was used to define tumor cellularity which was compared between low and high grade glioma and correlated with the glioma proliferative index.

**Results:**

The IDSI-derived cellularity values were elevated in both FLAIR and AMT-PET-derived regions of high-grade gliomas. ROC curve analysis found that the IDSI-derived cellularity can provide good differentiation of low-grade from high-grade gliomas (accuracy/sensitivity/specificity of 0.80/0.80/0.80). . Both apparent diffusion coefficient (ADC) and IDSI-derived cellularity showed a significant correlation with the glioma proliferative index (based on Ki-67 labeling; R = 0.95, *p* < 0.001), which was particularly strong when the tumor regions were confined to areas with high tryptophan uptake excluding areas with peritumoral edema.

**Conclusion:**

IDSI-MRI combined with AMT-PET may provide a multi-modal imaging tool to enhance pretreatment assessment of human gliomas by evaluating tumor cellularity and differentiate low-grade form high-grade gliomas.

**Electronic supplementary material:**

The online version of this article (doi:10.1186/s40644-015-0045-1) contains supplementary material, which is available to authorized users.

## Background

Malignant gliomas, including WHO grade IV glioblastomas, are among the most lethal malignancies in the human brain [1, 2]. The ability to distinguish actively proliferating neoplastic cells from non-tumoral lesions is critical to the clinical management of glioma patients. Current clinical neuroimaging techniques, including T1-weighted images with gadolinium (GAD) and fluid-attenuated inversion recovery (FLAIR), do not accurately differentiate regions with proliferating tumor cells from vasogenic edema and necrosis, and they are also inaccurate in predicting glioma grade and proliferative activity [3]. Thus, precise surgical planning for tailored tumor resection is limited based on current conventional MRI techniques. Advanced imaging techniques, including perfusion and diffusion MRI as well as positron emission tomography (PET) are being actively investigated to overcome some of the limitations of conventional MRI modalities [3].

Infiltrating gliomas often show high amino acid uptake extending beyond the contrast-enhancing tumor mass indicating glioma cell-infiltrated brain tissue [4, 5]. In clinical studies, several different amino acid PET radiotracers, including [^11^C]methionine, ^18^F-fluoroethyl-tyrosine (FET), ^18^F-fluoro-Ldihydroxy-phenylalanine (FDOPA), and α[^11^C]methyl-L-tryptophan (AMT) have been tested for glioma imaging, and each of them has their unique advantages and limitations [6]. Our center has recently tested the clinical use of AMT-PET for glioma imaging [6–11]. AMT is transported to tumor tissue via the large neutral amino acid transporter and can be metabolized via the immunomodulatory kynurenine pathway [6, 8, 12], whose activation leads to tumoral immune tolerance [13]. In clinical studies, increased AMT uptake was found to be useful to: (i) detect both low- and high-grade gliomas and identify glioma-infiltrated brain in non-enhancing brain regions (verified by histology) [5–7]; (ii) accurately differentiate recurrent gliomas from radiation injury [9]; and (iii) predict post-treatment survival in malignant gliomas [11]. However, PET has limitations, including relatively low spatial resolution and also limited clinical availability and high cost as compared to MRI techniques.

Recently, restricted diffusion measured by the apparent diffusion coefficient (ADC) on diffusion weighted imaging (DWI) was compared to postmortem cell density obtained by *ex vivo* histology of high-grade gliomas [14]. This comparison showed significantly increased cell density in the regions of low ADC (<0.929 × 10^−3^ mm^2^/s) and co-localized with FLAIR hyperintensity. However, DWI-derived ADC measures the average diffusion of water molecules within each voxel, limiting the differentiation of active tumor from necrosis [14]. Recent comparative studies of DWI-ADC and FDOPA, MET and FET PET showed that areas with restricted diffusion (low ADC) did not correlate with foci of high amino acid metabolism in human gliomas [15–17]. This discrepancy indicated that restricted diffusion may be affected not only by tumor cell density and metabolic activity but also other factors, such as ischemia or compression.

In order to detect subtle changes within voxels due to axonal injury, dense cellularity, demyelination, and edematous fluid in a mouse model of multiple sclerosis, diffusion basis spectrum imaging (DBSI) was developed. This technique can resolve multiple anisotropic and a spectrum of isotropic diffusion tensors in high angular resolution diffusion imaging (HARDI), by sampling water diffusion at 99 encoding directions and multiple diffusion weighting b-values [18]. It was reported that axonal water diffusion is distributed in non-Gaussian and the resulting signal decay is non-monoexponential requiring at least three b-values [19]. However, due to the long scanning time required for multi-b value HARDI acquisition, translation of this method to clinical practice is difficult, especially in patients with brain tumors routinely requiring several MRI scans for pre-treatment diagnosis and post-treatment monitoring.

The present study investigates whether the DBSI technique can be implemented for clinically feasible diffusion tensor imaging (DTI) studies which have been widely used for axonal tractography analysis to guide presurgical planning of tumor resection. It is heuristically presumed that independent component analysis with ball and stick model (ICA + BSM) [20] may provide accurate initial parameters of isotropic diffusion spectrum imaging (IDSI) [18] to assess tumor cellularity in clinical DTI data. The aim of the present study was to evaluate IDSI with ICA + BSM for clinical DTI data, specifically, to detect the degree of tumor cellularity in human gliomas. We hypothesized that malignant gliomas would show *increased* cellularity (i.e., high fraction of isotropic compartment at a low band of the diffusivity spectrum), as a result of *increased* restricted isotropic diffusivity within the active tumor region. We used both AMT PET and histopathology to evaluate the metabolic and cell proliferation marker correlates of increased cellularity defined by this novel approach.

## Methods

### Subjects and data acquisition

The study included ten patients with histopathologically-verified WHO grade II (*n* = 5) and grade IV (*n* = 5) gliomas (Table 1). The study was approved by Wayne State University’s Institutional Review Board and written informed consent was obtained from all participants.

**Table 1:** Patient demographics and tumor characteristics

Pt.	Age(years)	Gender	Tumor location	Glioma grade	Tumor proliferative index (%)	MRI max. tumor volume (10^−3^mm^3^)	Tumor/cortex ratio of AMT-SUV
1	70	F	Lt P	IV	50 %	53.8	1.7
2	54	F	Rt P	IV	25-30 %	103.3	1.9
3	37	F	Lt F	II	5-7 %	13.5	1.6
4	34	F	Rt P	II	2-3 %	1.5	1.8
5	70	F	Rt T-P	IV	20-25 %	24.6	2.0
6	78	M	Lt T	IV	30 %	50.7	2.2
7	45	M	Rt T	II	5 %	3.6	1.9
8	47	M	Lt T	IV	10-15 %^a^	42.7	1.6
9	18	M	Rt F	II	1-2 %	4.0	1.1
10	30	M	Lt T-F	II	5 %	2.4	2.4

*Pt*. patient, *F* female, *M* male, *Lt* left, *Rt* right, *F* frontal, *T* temporal, *P* parietal, *AMT*= α[^11^C]methyl-L-tryptophan, *SUV* standardized uptake value

^a^This glioma had evidence of intratumoral hemorrhage and widespread necrosis on histopathology

All MRI scans were performed on a 3T Philips scanner equipped with an eight-channel head coil. For IDSI, DTI data were gathered at repetition time (TR) = 10,870 ms, echo time (TE) = 108.9 ms, field of view (FOV) = 224 cm, 128 × 128 acquisition matrix, 2 mm thickness using 15 isotropic gradient directions with b-value = 1000 s/mm^2^, one b = 0 acquisition, and number of excitations = 1. For ADC, DWI data were acquired at 2 b-values of 0 and 1000 s/mm^2^ with all other imaging parameters the same as described above for IDSI. As a part of the clinical MRI protocol, conventional imaging sequences were acquired including axial pre- and post-contrast T1-weighted as well as T2-weighted and fluid attenuation inversion recovery (FLAIR) images.

AMT-PET images were acquired using a Siemens EXACT/HR whole-body positron emission tomograph (Siemens Medical Systems, Knoxville, TN), as described previously [5, 7, 9]. At 25 min after tracer injection, a dynamic emission scan of the brain (7 × 5 min) was started. Measured attenuation correction, scatter, and decay correction were applied. All images were reconstructed with filtered backprojection using a Hanning filter, yielding images with an in-plane resolution of 7.5 ± 0.4 mm at full-width half-maximum, and 7.0 ± 0.5 mm full-width half-maximum in the axial direction. For the present study, image analysis was performed using AMT standardized uptake value (SUV) images. The SUV was calculated by dividing the average tracer concentration in tissue at 30–55 min by the ratio of injected activity and patient weight. Regions with increased AMT SUV in the lobes encompassing the tumor were defined as areas showing at least 36 % higher AMT SUV as compared to mean AMT SUV in contralateral normal cortex. This threshold was established in our previous study [5] where this cutoff value provided an optimal pretreatment differentiation between the metabolically active tumor volume and peritumoral edema. In the same study, histopathology of stereotactically-resected brain tissue samples also verified that AMT-positive regions outside the contrast-enhancing tumor mass detected glioma-infiltrated brain when using this cutoff threshold [5].

**Fig. 1:**
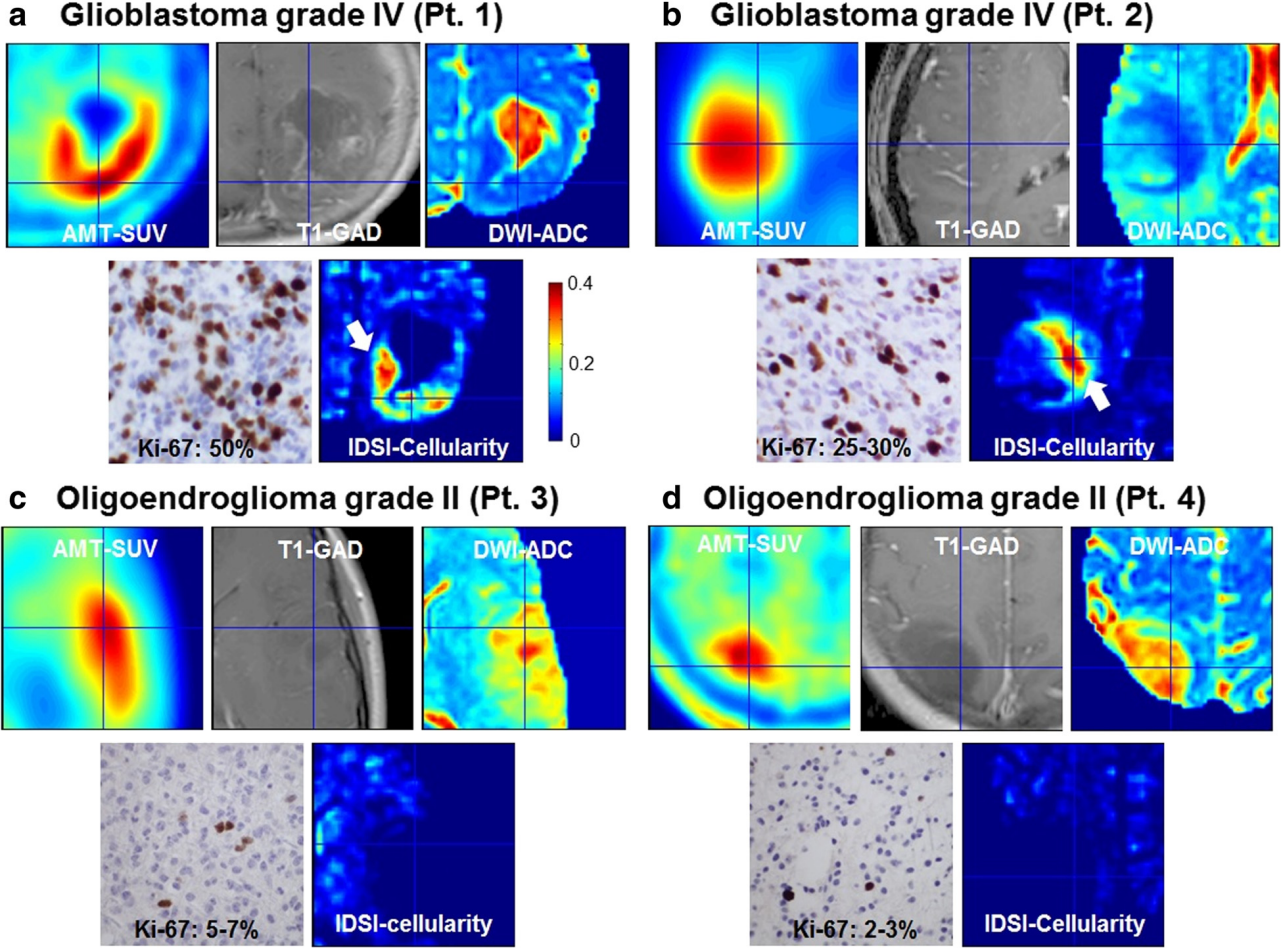
Representative images of AMT-SUV, T1-GAD, DWI-ADC and IDSI-cellularity obtained from two patients with a grade IV glioma (**a** and **b**), and two patients with a grade II glioma (**c** and **d**). The tumor in Pt. 1 (**a**) showed contrast enhancement surrounding a necrotic core on MRI. AMT-PET showed high uptake (in red) in the tumor region that surrounded the necrotic core with no AMT uptake; on histopathology, this tumor had dense cellularity and high Ki-67 labeling index in the region showing high AMT uptake (up to 50 % of the nuclei, as illustrated by the immunostaining on the bottom left). The tumor of Pt. 2 (**b**) showed minimal contrast enhancement on MRI, moderate cellularity and lower Ki-67 labeling index (25-30 % of the nuclei). Increased AMT-SUV showed the extent of the tumor with no necrotic core. White arrows indicate the cluster of voxels showing increased cellularity corresponding to increased AMT-SUV and decreased ADC value. Note that none of the voxels show high cellularity in the region of increased AMT-SUV in patients with a low-grade glioma (Pt. 3 and 4), consistent with low proliferative index and lower cellularity on histopathology, despite moderately high AMT uptake in these tumors

Standard neuropathological examination of tumor specimens was performed by an experienced board-certified neuropathologist (W.J.K.). Immunohistochemical staining for Ki-67 (monoclonal antibody, Dako, Santa Barbara, CA) was performed to assess glioma proliferative activity. The Ki-67 labeling index was determined by identifying the areas of greatest tumor cellularity, examining at least 10 high-power fields (40×) on an Olympus microscope, and determining the ratio between the number of Ki-67-positive tumor cell nuclei and the total number of tumor cell nuclei in each high-power field (Fig. 1). The proliferation index was expressed as a percent range (lowest to highest, e.g., 5-10 %). Small, isolated fields with higher Ki-67 labeling that were not reflective of the entire tumor mass were excluded from the analysis.

### Assessment of tumor cellularity from clinical DTI data

The present study utilized an ICA + BSM analysis [20] to facilitate the numerical procedure of model selection and non-linear optimization in a conventional IDSI framework [18] (e.g., details are available in Additional file 1). In brief, using IDSI fitting analysis constrained by ICA + BSM, voxel-wise isotropic diffusion fractions, f_dk=1,2,3,4_ were estimated at four discrete isotropic spectral bands, d_1_ = 0, d_2_ = 1.0, d_3_ = 2.0 and d_4_ = 3.0 × 10^−3^ mm^2^/s. To assess levels of the cellularity, we defined IDSI-cellularity value at every voxel by summing two low spectral fractions (i.e., cellularity = f_d1_ + f_d2_). The upper bound of the fraction (d_k_ = 1.0 × 10^−3^ mm^2^/s) was selected according to maximal ADC threshold (0.929 × 10^−3^ mm^2^/s) measured for the cutoff value for hypercellularity previously validated by histology [14].

For the voxel-wise comparison of individual patients, T1-GAD, FLAIR, DWI-ADC and AMT-SUV, images were co-registered to the b0 image applying the corresponding affine transformation that optimizes the cost function of normalized mutual information embedded in SPM 8 software (http://www.fil.ion.ucl.ac.uk/spm/).

### Performance assessment of IDSI-derived cellularity to differentiate high-grade from low-grade gliomas

Receiver operating characteristic (ROC) curve analysis was utilized to assess the accuracy of the DWI-ADC and the IDSI-derived cellularity to differentiate high-grade (grade IV, n = 5) from low-grade gliomas (grade II, *n* = 5) in two, differently defined tumoral regions of interest (ROI), 1) FLAIR ROI, which manually delineated to include hyperintense regions in the area of the presumed tumor on FLAIR images by including all tumor regions showing higher intensity than normal contralateral cortex, and 2) AMT-SUV ROI including voxels with high AMT-SUV above the 36 % threshold as compared to contralateral normal cortex, Y = tumor/cortex ration of AMT-SUV ≥ 1.36 [5]. In each of the two tumoral ROIs, IDSI-derived cellularity was measured to quantify the degree of cellularity in individual patients. The ROC curves were fitted using non-linear square fit of the measured true positive ratio (sensitivity) to the measured false positive rate (1-specificity). The area under curve (AUC) was then calculated from the fitted curve for the comparison.

### Correlation of glioma proliferative index with DWI-ADC and IDSI-derived cellularity

In order to assess IDSI-derived cellularity to predict tumor proliferative activity, we correlated mean glioma Ki-67 labeling index with the mean values of DWI-ADC and IDSI-derived cellularity obtained in both FLAIR and AMT-SUV derived tumor ROIs using Pearson’s correlations. For this analysis, median Ki-67 labeling index from nine patients were used. One patient with a grade IV glioma (Pt. 8) had evidence of intratumoral hemorrhage before surgery. Histopathology showed densely cellular tumor parts but an unusually low Ki-67 index for a grade IV tumor, due to extensive necrotic and hemorrhagic tissue damage. Therefore, in this case, Ki-67 labeling index was not an appropriate surrogate marker of tumor cellularity and was excluded from this analysis.

## Results

Representative examples of AMT-SUV, T1-GAD, ADC, and IDSI-derived cellularity $\left({\varSigma}_{{\mathrm{d}}_{\mathrm{k}}=0}^{{\mathrm{d}}_{\mathrm{k}}=0.0010}{\mathrm{f}}_{{\mathrm{d}}_{\mathrm{k}}}\right)$ are shown for two patients with a grade IV glioma and two patients with a grade II glioma (Fig. 1). As marked by white arrows, the IDSI-derived cellularity detected a cluster of voxels in the region showing increased AMT-SUV and decreased ADC values in both grade IV gliomas. In contrast, no voxels with increased cellularity was found in either grade II gliomas, which showed lower cellularity and low Ki-67 labeling indices (up to 7 %) on histopathology. This suggests that the proposed IDSI-derived cellularity measure may provide an effective tool to identify tumors with high proliferative activity.

**Fig. 2:**
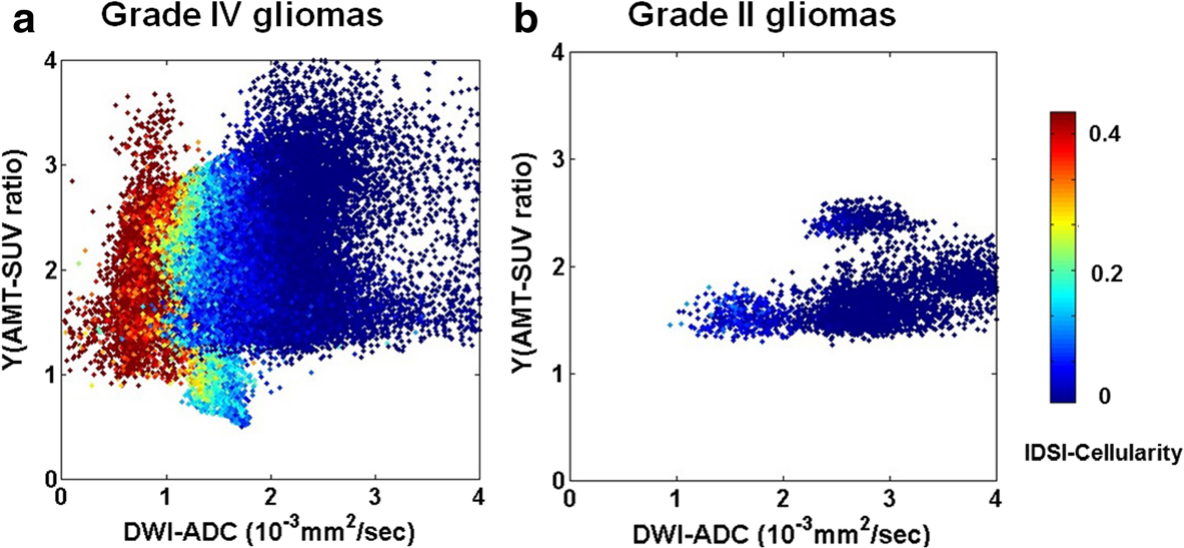
Scatter plots of the IDSI-derived cellularity compared with Y(tumor/cortex ratio of AMT-SUV) and DWI-ADC values obtained from FLAIR-derived tumor regions of five patients with grade IV gliomas (**a**), and five patients with grade II gliomas (**b**). Each plot shows the summation of individual voxels for which DWI-ADC and Y(AMT-SUV ratio) are plotted on the x- and y-axes, respectively, while cellularity values are indicated by color. Note that increased cellularity tends to occur in voxels with low ADC values for all grade IV glioma cases, while there were much fewer voxels with increased cellularity in the grade II gliomas

**Fig. 3:**
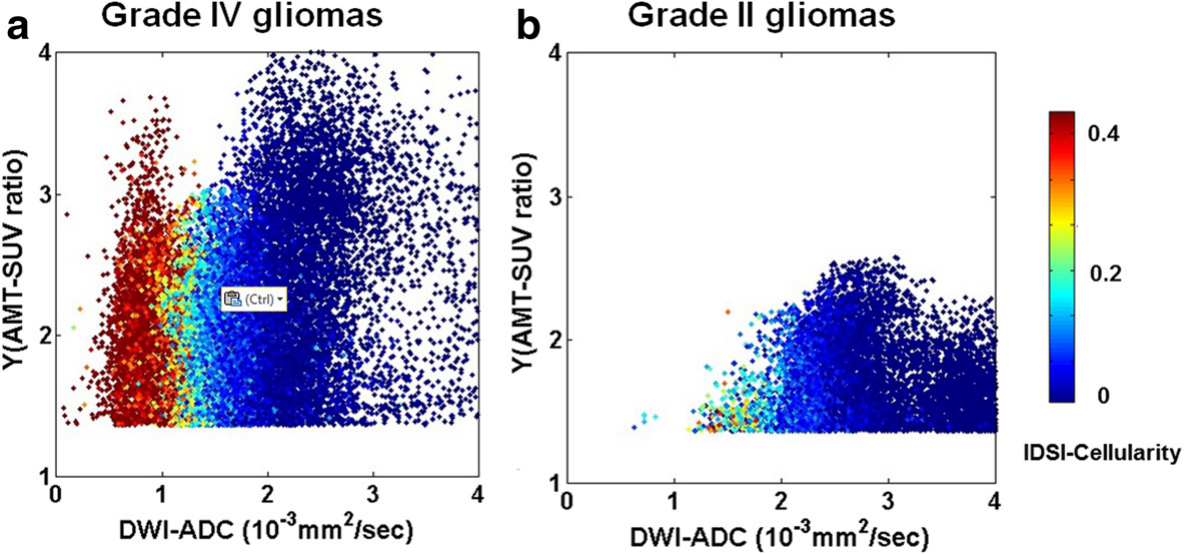
Scatter plots of the IDSI-derived cellularity compared with Y(tumor/cortex ratio of AMT-SUV) and DWI-ADC values obtained from AMT-PET-derived tumor regions of five patients with grade IV gliomas (**a**), four patients with grade II gliomas (**b**). Pt. 10 excluded from grade IV glioma since no tumor/cortex ratio was found above the threshold of 1.36 (Y ≥ 1.36). Each plot represents an individual voxel for which DWI-ADC and Y(AMT-SUV ratio) are plotted on the x- and y-axes, while cellularity values are indicated by color. Note that increased cellularity tends to occur in voxels with low ADC values for all grade IV glioma cases, and also that there were no voxels with increased cellularity in the grade II gliomas

Figure 2 shows scatter plots of IDSI-derived cellularity obtained from FLAIR ROIs of five patients with grade IV gliomas and five patients with grade II gliomas. The color of each dot indicates the value of IDSI-derived cellularity for each voxel of the tumor region. Increased IDSI-derived cellularity was apparent in the voxels with higher AMT-SUV and lower ADC in high-grade glioma patients. Similarly, Fig. 3 represents scatter plots of IDSI-derived cellularity obtained from AMT-SUV ROIs of five patients with grade IV gliomas and four patients with grade II gliomas (in one grade II tumor, AMT-SUV increase did not exceed the 36 % cutoff threshold (i.e., Y ≥ 1.36), and this tumor was not included in this analysis). Compared with plots obtained in FLAIR-based ROIs (Fig. 2), increased IDSI-derived cellularity was more apparent in the voxels with higher AMT-SUV and lower ADC only in high-grade glioma patients, while low-grade gliomas showed no voxels with high cellularity.

**Fig. 4:**
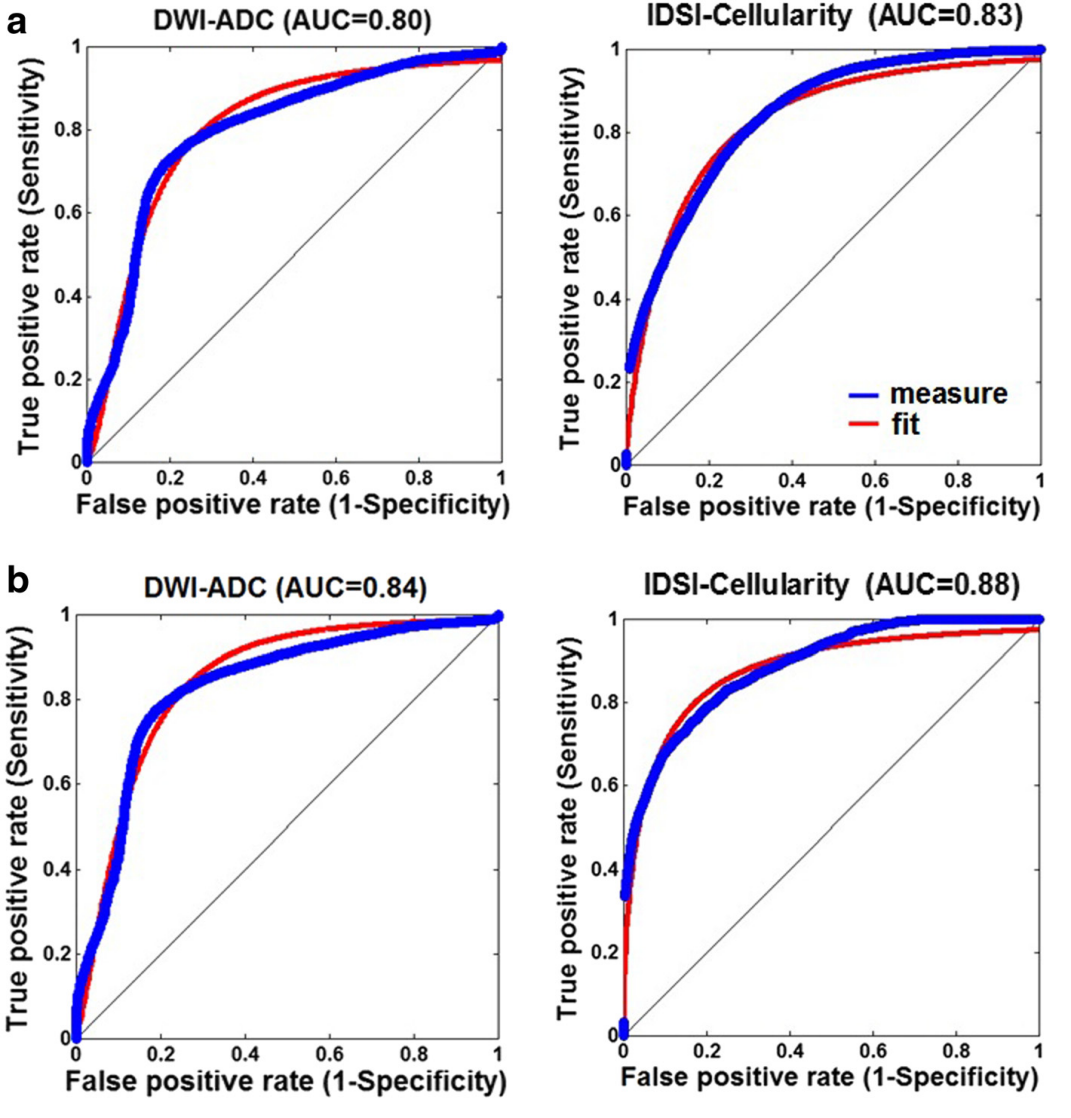
ROC curves (blue: measured, red: fitted) obtained from FLAIR-based tumor regions (**a**) and high AMT-SUV tumor regions (**b**) in order to differentiate grade IV gliomas from grade II gliomas using DWI-ADC and IDSI-cellularity. In each curve, true positive rate (y-axis) was estimated using non-linear least square fit of the false positive rate (x-axis) using the equation of y = 1 − 1/(1 + (x/a)^b^)^c^ where a, b and c are the model coefficients. For the comparison, the area under curve (AUC) was finally calculated from the fitted curve

In differentiating high-grade to low-grade gliomas, compared with the DWI-ADC, the IDSI-derived cellularity showed a slightly higher probability to differentiate high grade from low grade glioma, resulting in ‘good test’ for both FLAIR and AMT-SUV tumoral ROIs (Fig. 4, AUC = 0.83 and 0.88 for FLAIR and AMT-SUV).

**Fig. 5:**
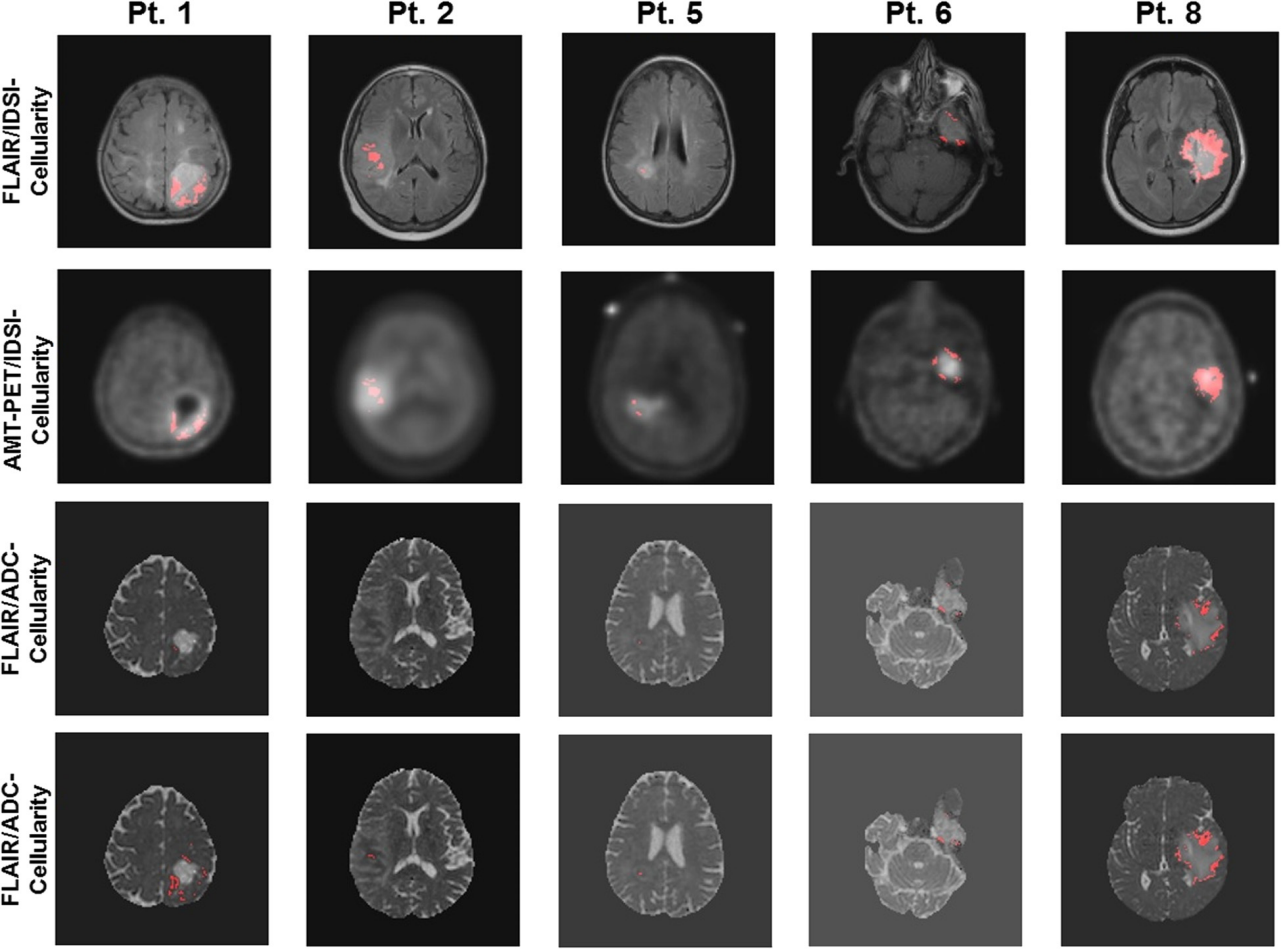
Receiver operating characteristic (ROC) curve analysis was applied to estimate the optimal cut-off value of IDSI-cellularity (0.17), yielding the accuracy/sensitivity/specificity of 0.80/0.80/0.80 to differentiate grade IV gliomas from grade II gliomas. The detection of the proliferating tumor cells was performed by thresholding of IDSI-derived cellularity at its cut-off value. Red-colored voxels survived the cutoff threshold inside the FLAIR hyperintense tumor regions (1^st^ row) and the AMT high uptake regions (2^nd^ row). For the comparison, the detection of the proliferating tumor cells inside the FLAIR hyperintense tumor regions was performed by thresholding of DWI-ADC image at two cut-off values, 0.93 × 10^−3^ mm^2^/s (3^rd^ row) and 1.22 × 10^−3^ mm^2^/s (4^th^ row) which were reported as the ADC values for hypercelluarity previously validated by histology [14, 21]

Delineation of the tumor area with high cellularity was performed by thresholding the IDSI-derived cellularity map at its optimal cut-off value (0.17) in both FLAIR and AMT-SUV tumoral ROIs, yielding the accuracy/sensitivity/specificity of 0.80/0.80/0.80 to differentiate the voxels of grade IV from those of grade II in the ROC analysis (Fig. 5). For the comparison with DWI-derived ADC cellularity, previously histology-validated maximal ADC thresholds, 0.93 × 10^−3^ mm^2^/s [14] and 1.22 × 10^−3^ mm^2^/s [21] were applied to define the active tumor areas inside hyper intense FLAIR regions of the same patients. The IDSI-derived cellularity detected larger proliferating tumor areas with high cellularity than the DWI-derived ADC cellularity. On the other hand, the high-cellularity areas were smaller than the areas showing high AMT SUV in all patients with grade IV glioma.

**Fig. 6:**
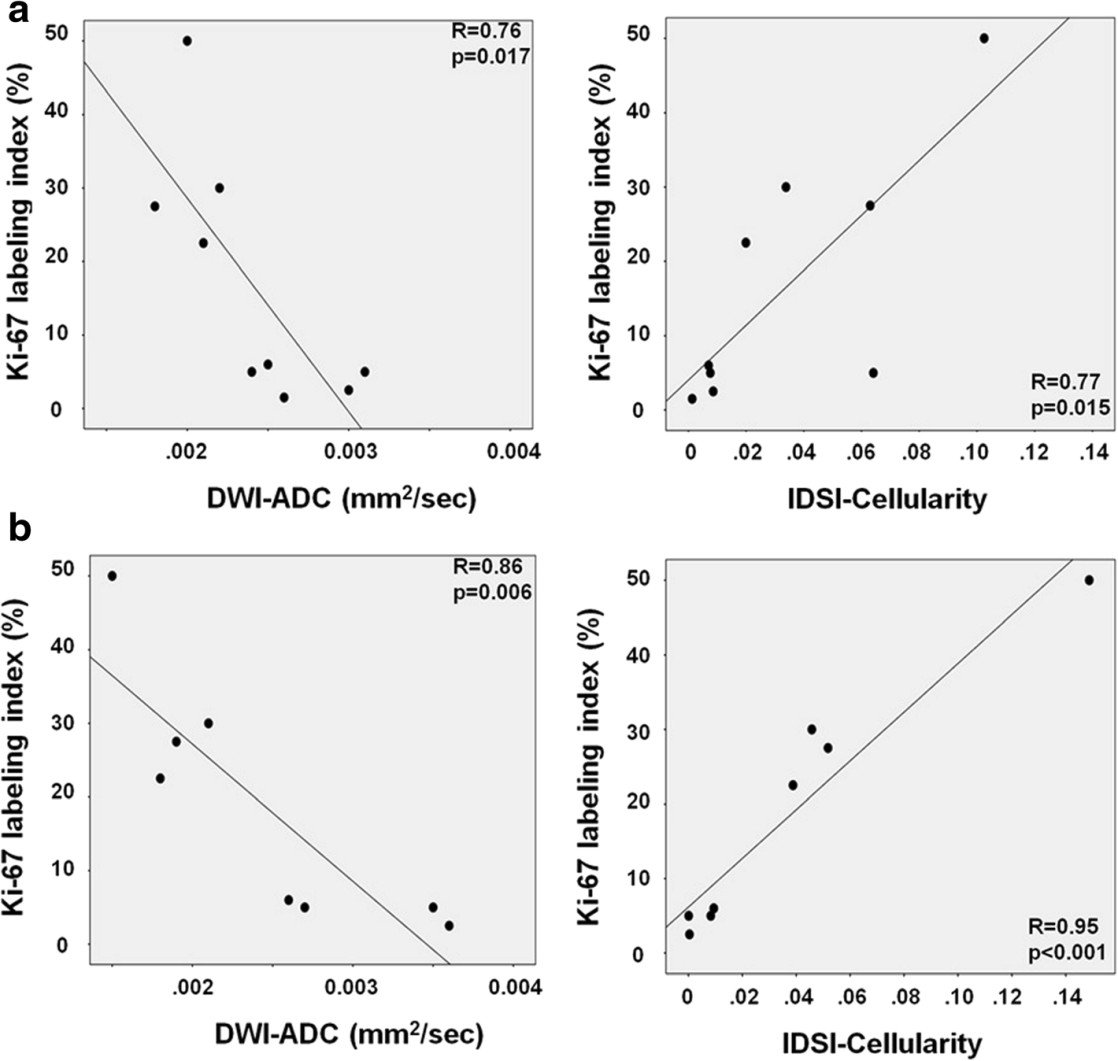
Close correlation of glioma Ki-67 labeling index with DWI-ADC (left) and IDSI-cellularity (right) obtained from the FLAIR ROI (**a**) and AMT-SUV-based ROI (**b**). The correlation coefficients and p values are based on Pearson’s correlations

IDSI-derived cellularity correlated positively with Ki-67 labeling index when tested using the FLAIR-based tumor ROIs(R = 0.77, *p* = 0.015, Fig. 6a). The correlation was even stronger when using the AMT-PET-based tumor ROIs (R = 0.95, *p* < 0.001, Fig. 6b). This is not surprising, because the AMT-SUV ROIs include the active tumor regions, while T1-FLAIR ROI can include vasogenic edema. Because Ki-67 values are derived from tumor tissue but not from tissue with edema, the correlation is stronger with the PET-based regions.

## Discussion

The present study demonstrates the potential clinical use of IDSI-derived cellularity as a new measure for clinically feasible DTI studies to assess hypercellularity in malignant gliomas. In grade IV gliomas, high IDSI-derived cellularity values were found in portions of both areas showing increased FLAIR signal intensity, which can include both tumor and peritumoral edema, and in regions with increased AMT-SUV, a PET imaging marker of glioma mass and glioma-infiltrated peritumoral brain parenchyma [5, 7]. In these regions, the cellularity parameter had a high accuracy to differentiate grade IV from grade II gliomas. By adapting the isotropic diffusion spectrum to measure restricted isotropic diffusivity in the framework of ICA + BSM, our data also suggest that this technique can provide a novel imaging marker to estimate glioma proliferative activity while using a clinically feasible DTI acquisition.

In the literature, there is disagreement regarding the interpretation of DWI-derived ADC changes in brain tumors. Several studies [22–26] reported an increased tumor cellularity in the region showing decreased ADC values, while other studies [27, 28] showed necrosis in the regions of low ADC. Also, DWI-derived ADC is sensitive to other tissue characteristics, including the presence of extracellular fluid due to vasogenic edema or tumor-induced destruction of the extracellular structure. In addition, gliomas often contain a variable number of infiltrating immune cells and microglia [29, 30], which can be numerous in high-grade gliomas and may affect the measured diffusion characteristics. Therefore, it is unclear how useful restricted diffusion could be to indicate “hypercellularity” to identify tumor-infiltrated brain and localize such regions outside of the contrast-enhancing tumor mass.

In the present study, AMT-PET was combined with DTI images to determine whether IDSI-MRI could be used to detect active tumor regions while excluding areas with necrosis and vasogenic edema, which show very low AMT uptake [5]. The results show that such detection can be done with great accuracy in high-grade gliomas, which always show a marked increase of AMT-SUV [7]. In these regions, the IDSI approach detected increased cellularity consistent with the tumors’ strong proliferative activity and dense cellularity, as demonstrated on histopathology (Fig. 1). In contrast, all low-grade gliomas showed relatively low cellularity values by IDSI, despite the variable increase of AMT uptake on PET. Low-grade gliomas have relatively low proliferative activity and typically show low glucose metabolism on PET. Still, the majority of low-grade gliomas show increased AMT uptake and trapping, which can be attributed to increased tryptophan transport and metabolism via the kynurenine pathway that can occur in both low- and high-grade gliomas [8, 12]. Therefore, tumoral AMT uptake measures are generally not very accurate to predict glioma grade and proliferative activity. Our results are consistent with this and show that IDSI could provide a better differentiation between low-grade and high-grade gliomas by the estimation of tumor cellularity.

Our preliminary data also demonstrate that IDSI is a promising imaging modality for the non-invasive estimation of glioma proliferative activity, as suggested by the correlation between IDSI-derived cellularity and Ki-67 labeling index (Fig. 6). The correlation appeared to be stronger in PET-derived tumor ROIs (Fig. 6b) than in FLAIR-based ROIs, likely because the FLAIR-based ROIs included non-tumorous edema in some cases (Fig. 6a). In contrast, the PET-based ROIs included tumor portions with high tryptophan metabolic activity but excluded peritumoral vasogenic edema where proliferative activity is low. However, PET-based ROIs cannot be used in some low-grade gliomas showing low AMT uptake, as was seen in one of our cases. Also, malignant gliomas are often heterogenous and can contain tumor portions showing different histologic grade and proliferative activity, which may have similar high AMT uptake on PET. Comparison of AMT-SUV and IDSI cellularity maps (Fig. 5) suggests that IDSI may be able to identify glioma subregions with the highest cellularity, which may also be the most proliferative areas. This could be tested in future studies by stereotactic, image-guided sampling targeting tumor regions showing different cellularity values.

Since AMT-PET and IDSI characterize different tumor characteristics, the two imaging modalities may also provide complementary information in pre-treatment evaluation of gliomas. IDSI may provide a parameter more specific for tumor cellularity and less affected by other factors such as tumoral ischemia or mass effects. On the other hand, AMT is a unique PET tracer because its tumoral accumulation is not only a reliable marker of active glioma tissue but can also detect inflammatory cell infiltration [31]; while necrotic regions of brain tumors, as well as pure, glioma-induced vasogenic edema show little to no AMT accumulation [5]. Thus, when IDSI-MRI is applied in the region of high AMT uptake, it becomes possible to evaluate cellularity due to tumor cell proliferation and immune cell infiltration in active tumor regions without including either necrosis or edema.

Other parameters derived from IDSI-MRI may also be able to provide useful information by detecting such necrotic and edematous regions, which likely show high fluid ratio indicating the presence of increased extracellular fluid due to the damaged membrane architecture. In the current study, we focused on the assessment of tumor cellularity, but future studies could evaluate other IDSI-MRI-derived parameters (characterizing fluid ratio, demyelination or axonal injury) to determine if they provide additional clinically useful information in glioma evaluation.

There are several potential limitations in this study. First, non-simultaneous acquisition of AMT PET and MRI may lead to potential sources of errors in registering locations of increased AMT and IDSI-defined cellularity. This limitation could be overcome by the use of integrated PET/MR systems able to simultaneously acquire MR and PET images. Alternatively, tumor delineation may be performed using FLAIR images; such a region can include some peritumoral edema, but this approach is more feasible clinically and does not require the use of PET images. Secondly, due to the small number of diffusion encoding directions in clinical MRI data, that may provide insufficient sensitivity for cellularity, we calculated cellularity by summing the fractional ratios of isotropic diffusion at the spectral range of 0–1.0 × 10^−3^ mm^2^/s. The present study adopted the cellularity threshold of spectral band (1.0 × 10^−3^ mm^2^/s) from the previous postmortem study [14] which reported the ADC value of 0.93 × 10^−3^ mm^2^/s as a maximal ADC value of high grade gliomas. However, as presented in Fig. 5, the thresholding of ADC map at this threshold resulted in much less and sparser tumoral areas rather than at higher ADC threshold (1.22 × 10^−3^ mm^2^/s), which was reported as an average ADC values of high grade tumor in pediatric brain [21]. The present study could not guarantee the effect of higher spectral band threshold on the size of tumoral area since pathological information is not available at every tumoral area. This ambiguity of actual IDSI threshold may cause a significant variation in cellularity across different histologic grades of gliomas. Thus, future studies should validate the results of this study in larger cohorts with detailed pathology including grade II, III, and IV gliomas.

## Conclusions

By using IDSI to assess hypercellularity in newly-diagnosed gliomas, we found that both FLAIR-defined and PET-defined active tumor regions contain greater IDSI-derived cellularity in high-grade gliomas compared to low-grade gliomas. Tumoral cellularity values showed a close correlation with the glioma proliferative index as defined by histopathology. These initial results hold the promise of a new, refined imaging tool to better characterize glioma cellularity and differentiate glioma grades using clinically applicable MRI acquisitions.

## Additional file

Additional file 1:
**Isotropic diffusion spectrum imaging (IDSI) with independent component analysis with a ball and stick model.** (DOCX 20 kb)
