# Supplementary material for: Multi-modal imaging of tumor cellularity and Tryptophan metabolism in human Gliomas
Source: Cancer Imaging. 2015 Aug 6;15(1):10. doi: 10.1186/s40644-015-0045-1 (PMC4527188; doi:10.1186/s40644-015-0045-1)
Supplement: Additional file 1: — Isotropic diffusion spectrum imaging (IDSI) with independent component analysis with a ball and stick model. (DOCX 20 kb) [file 40644_2015_45_MOESM1_ESM.docx]

**Isotropic diffusion spectrum imaging (IDSI) with independent component analysis with a ball and stick model**

ICA+BSM was utilized to facilitate the numerical procedure of model selection and non-linear optimization in a conventional IDSI framework: the *j^th^*-diffusion-gradient measurement S_j_ is considered as a linear combination of *K*-anisotropic and a spectrum of isotropic diffusion compartments [18],

$S_{j}=\sum_{k=1}^{K} f_{k}e^{-\left| \vec{b}_{j} \right|\lambda_{\perp k}}e^{-\left| \vec{b}_{j} \right|{(\lambda}_{\parallel k}{-\lambda}_{\perp k})\cos^{2}\theta_{\mathrm{kj}}}+\int_{0}^{D_{\max}} f\left( D \right)e^{-\left| \vec{b}_{j} \right|D}\mathrm{dD}$ [1]

where the first term models the *K*-crossing axonal bundles in white matter tissue characterized by the following four parameters of cylindrically symmetric tensors,$\lambda_{\parallel k}$: axial diffusivity, $\lambda_{\perp k}$: radial diffusivity, f_k_: intensity fraction, and $\theta_{\mathrm{kj}}$: the angle between the *j^th^* diffusion gradient and the principal direction of the *k^th^* anisotropic diffusion tensor. The second term is to model restricted isotropic water diffusion in cells, sub-cellular structure, and edematous tissue characterized by f(D): isotropic diffusion spectrum and D_max_: high diffusivity limit. $\left| \vec{b}_{j} \right|$ is the b-value of the j^th^ diffusion gradient, j=1,2,...,*N* which is the total number of encoding gradients.

The model complexity of Eq. 1 is dramatically increased depending on the number of crossing axons, *K_opt_* and heterogeneity of white matter tissue, which typically force the convergence of non-linear optimization to local solutions [16]. In the implementation of IDSI with clinical data sampling water diffusion at low encoding gradients, a two-step approach was employed to solve Eq. 1. In the first step, three key parameters of Eq. 1: f_k_,$\theta_{\mathrm{kj}}$, and *K* were estimated in the framework of ICA+BSM which assumes that the measured S_j_ is a linear summation of a single isotropic diffusion tensor (**D**_0_, mean offset of the ball compartment reconstructed by whitening and de-whitening of a set of diffusion data measured at a local cluster) and *K_opt_*- directionally independent diffusion tensors (**D**_k_, uncorrelated-zero-mean-unit-variance diffusion signals of K_opt_-stick compartments).

$S_{j}\cong\sum_{k=1}^{K_{\mathrm{opt}}} f_{k}e^{-\left| \vec{b}_{j} \right|r_{j}^{T}D_{j}r_{j}}+(1-\sum_{k=1}^{K_{\mathrm{opt}}} f_{k})e^{-\left| \vec{b}_{j} \right|r_{j}^{T}D_{0}r_{j}}$ [2]

where f_k_ is the volume fraction of the *k*^th^ stick compartment, characterized by the diffusion tensor **D***_k_*(= **E***_k_***V***_k_***E***_k_*^T^, **E***_k_* =[e_k1_ 0 0; e_k2_ 0 0; e_k3_ 0 0], and **V***_k_* denote eigenvector matrix with the fixed eigenvalue matrix, diag(0.0017 mm^2^/s, 0, 0), of tensor matrix **D***_k_*, respectively). **D**_0_ represents the diffusion tensor of isotropic diffusion parameterized by diag(λ, λ, λ). **r***_j_* is the unit vector of the *j^th^* diffusion gradients. The superscript of T stands for the transpose. Under the equality constraint of $\sum_{k=1}^{K_{\mathrm{opt}}} f_{k}=1$, the ICA decomposition is performed to seek *K_opt_*-independent diffusion profiles mixed in a local measurement. The mixing ratios and the principal direction of the Gaussian tensors of the identified *K_opt_*-profiles are then utilized as initial estimates of f_k_ and **E**_k_ =[e_k1_ 0 0; e_k2_ 0 0; e_k3_ 0 0] in the least square fit. The overall fitting with random initialization of λ is repeated until an optimal solution of parameter sets {f_k_, λ, e_k1_, e_k2_, e_k3_, *K_opt_*} is achieved in the framework of Bayesian information criteria [19].

In the second step, the optimal solution of ICA+BSM ({f_k_, e_k_, *K_opt_*} in Eq. 2 was utilized to simplify the numerical model of IDSI into a linear summation of *K_opt_*-anisotropic and a discrete spectrum of *L*-isotropic diffusion compartments.

$S_{j}\cong\sum_{k=1}^{K_{\mathrm{opt}}} f_{k}e^{-\left| \vec{b}_{j} \right|\lambda_{\perp k}}e^{-\left| \vec{b}_{j} \right|{(\lambda}_{\parallel k}{-\lambda}_{\perp k})\cos^{2}(\cos^{-1} \frac{r_{j}\cdot e_{k}}{\left| r_{j} \right|\left| e_{k} \right|})}+\sum_{k=K_{opt+1}}^{K_{\mathrm{opt}}+L} f_{k}e^{-\left| \vec{b}_{j} \right|d_{k}}$ [3]

where a set of parameters {e_k_, *K_opt_*} is fixed according to the solution of Eq. 2 in the overall fitting process. The fractions of anisotropic components,{f_k=1,...,_*_Kopt_*} are initialized by the solution of Eq. 2 and then optimized with other parameters: $\lambda_{\parallel k}$, $\lambda_{\perp k}$, a spectrum of isotropic components, {f_k_=*K_opt+1_*,..,*K_opt+L_*} using generalized pattern search algorithm under the equality constraint of $\sum_{k=1}^{K_{opt+L}} f_{k}=1$. d_k_ indicates the discrete value of isotropic diffusivity equally sampled at *L*-bins ranging from 0 to 3.0×10^-3^ mm^2^/s.

**Extraction of isotropic diffusivity measures**

On the convergence of a generalized pattern search algorithm to the optimal minimum via multiple random initializations of {$\lambda_{\parallel k=1,\ldots,Kopt}$, $\lambda_{\perp k=1,\ldots,Kopt}$, f_k=1,...,_ *_Kopt_*_,...,_*_Kopt+L_*}, Eq. 3 can be fully implemented to create complete diffusivity metrics associated with intact axonal bundles (i..e, *K_opt_* ≤ 3), 1) the fractions of crossing axons (f_k=1,...,_*_Kopt_*), 2) anisotropic diffusivities of *K_opt_-*axonal components effectively quantifying axonal injury/loss ($\lambda_{\parallel k}$) and demyelination ${(\lambda}_{\perp k})$, and 3) isotropic diffusivity spectrum representing the fractions of stationary diffusion compartments highly restricted inside cells or other sub-cellular structures (i.e., f_k=_*_Kopt+1_*_,...,_*_Kopt+L_* where f_k=_*_Kopt+1_* and f_k=_*_Kopt+L_* indicate the fractions of isotropic diffusivity components measured at d_k_=0 and 3.0×10^-3^ mm^2^/s, respectively).
